# Activated Sludge Microbial Community and Treatment Performance of Wastewater Treatment Plants in Industrial and Municipal Zones

**DOI:** 10.3390/ijerph17020436

**Published:** 2020-01-09

**Authors:** Yongkui Yang, Longfei Wang, Feng Xiang, Lin Zhao, Zhi Qiao

**Affiliations:** 1 School of Environmental Science and Engineering, Tianjin University, Tianjin 300350, China; ykyang@tju.edu.cn (Y.Y.);; 2 China-Singapore Joint Center for Sustainable Water Management, Tianjin University, Tianjin 300350, China

**Keywords:** activated sludge, industrial zone, metabolic function, microbial community, wastewater treatment

## Abstract

Controlling wastewater pollution from centralized industrial zones is important for reducing overall water pollution. Microbial community structure and diversity can adversely affect wastewater treatment plant (WWTP) performance and stability. Therefore, we studied microbial structure, diversity, and metabolic functions in WWTPs that treat industrial or municipal wastewater. Sludge microbial community diversity and richness were the lowest for the industrial WWTPs, indicating that industrial influents inhibited bacterial growth. The sludge of industrial WWTP had low *Nitrospira* populations, indicating that influent composition affected nitrification and denitrification. The sludge of industrial WWTPs had high metabolic functions associated with xenobiotic and amino acid metabolism. Furthermore, bacterial richness was positively correlated with conventional pollutants (e.g., carbon, nitrogen, and phosphorus), but negatively correlated with total dissolved solids. This study was expected to provide a more comprehensive understanding of activated sludge microbial communities in full-scale industrial and municipal WWTPs.

## 1. Introduction

Activated sludge (AS) processes are the most widely used biological processes in wastewater treatment plants (WWTPs) worldwide, and they have been employed for pollutant removal for more than a century, owing to their high nutrient removal, toxin degradation, and biomass retention capabilities [1,2,3]. Microbial community structure and diversity affect the performance and functional stability of WWTPs [4,5,6]. Therefore, knowledge of AS microbial community structure and microbial functions will facilitate sludge population optimization and improve WWTP operation.

The Chinese government has heavily promoted industrial zone development, and there were 626 national and 1141 provincial zones in China, as of 2017. Reductions in common chemical oxygen demand (CODcr), ammonia–nitrogen (NH_4_-N), total nitrogen (TN), and total phosphorus (TP), via wastewater treatment in industrial zones located in the Haihe Water Basin, China, contributed to 26.2%, 23.9%, 20.3%, and 29.0%, respectively, of the total pollutant reduction achieved through municipal and industrial wastewater treatment in China [7]. This implies that treatment of wastewater generated by China’s industrial zones will facilitate water pollution control in China [8]. Factories sometimes discharge their wastewater without any effective pretreatment into centralized WWTPs of industrial zones. Industrial wastewater contains complex hazardous substances, such as heavy metals, terephthalic acid, phthalic acid, and benzoic acid, and therefore, has low biodegradability and is highly toxic [9,10].

Several conventional biomolecular techniques, such as denaturing gradient gel electrophoresis [1], terminal restriction fragment length polymorphism analysis [11], reverse transcription-polymerase chain reaction (RT-PCR) [12], and fluorescence in situ hybridization [13], have been widely used to determine bacterial species and functional genes of microbial communities. These methods are specific but of low throughput as they cannot provide a comprehensive profile of the bacterial community structure and function, due to amplification bias. Recently developed powerful and highly efficient high-throughput sequencing techniques, such as Illumina sequencing platforms and 454 pyrosequencing, provide enough sequencing depth and high accuracy for rapidly determining complex microbial communities and metabolic pathways [14,15]. However, knowledge about the microbial community in sludge during industrial wastewater treatment, especially from WWTPs in centralized industrial zones of different industries, remains limited.

The objective of this study was to determine the structure and diversity of sludge bacterial communities in two WWTPs in centralized industrial zones, as well as a municipal WWTP. This entailed the analysis of the pollutant removal capacities of different WWTPs, the microbial community structure of sludge, the key metabolic functions of different microorganisms, and the effects of influent wastewater characteristics and treatment process on bacterial communities. This study was expected to contribute towards a more comprehensive understanding of sludge microbial community structure in full-scale industrial and municipal WWTPs, which would in turn offer insight into achieving better pollutant removal performance of WWTPs.

## 2. Materials and Methods

### 2.1. WWTP Operation Conditions and Sampling

Three WWTPs, referred to as Y-, D-, and Z-WWTP, were considered in this study. Y-WWTP is a centralized WWTP in the chemical industrial zone Y located in the Tianjin Economic-Technological Development Area (TEDA). TEDA was established in 1984 as one of China’s first national-level industrial zones. It generates a gross domestic product of 305 billion yuan/year. Chemical industrial zone Y has 300 factories, including chemical, salt production, paper making, and machining factories. Y-WWTP employs an oxidation ditch (OD) with a treatment capacity of 10 × 10^4^ ton/d. This plant operates at a mixed liquid suspended solid (MLSS) content of 3850 mg/L, sludge retention time (SRT) of 24.5 d, hydraulic retention time (HRT) of 19.2 h, and dissolved oxygen (DO) content of 2.4 mg/L. Industrial wastewater accounts for 60% of the influent to this plant, of which 70% is wastewater from chemical industries. Its effluent meets level B of the Chinese discharge standard of pollutants for municipal WWTPs (GB 18918-2002).

D-WWTP is also a centralized WWTP in comprehensive industrial zone D, which has approximately 3000 industries covering communication, food processing, machinery, and biopharmaceuticals. The composition of the D-WWTP influent is 40% industrial wastewater and 60% local municipal wastewater. It employs a sequencing batch reactor (SBR)-based demand aeration tank and intermittent aeration tank with a treatment capacity of 9 × 10^4^ ton/d at an MLSS content of 3450 mg/L, SRT of 20.0 d, HRT of 4.5 h, and DO content of 3.0 mg/L.

Z-WWTP is a municipal WWTP in the Z district and employs an anoxic–oxic (A/O) process. It has a treatment capacity of 20 × 10^4^ ton/d at an MLSS content of 3500 mg/L, SRT of 25.0 d, HRT of 19.5 h, and DO content of 2.0 mg/L.

The influent and effluent of the WWTPs and AS in the aeration tanks were collected every three days from September (monthly mean temperature: 24.5 °C) to November (monthly mean temperature: 5.6 °C), during the dry weather in 2017. Analyses of pollutant concentration, pollutant removal from wastewater, and sludge microbial community are detailed in the following sections.

### 2.2. DNA Extraction, PCR Amplification, and High-Throughput Sequencing

Total genomic DNA was extracted from sludge samples (300-mg wet weight) using a PowerSoil DNA isolation kit (MO BIO Labs, Solana Beach, CA, USA). DNA quality and quantity were assessed through agarose gel (1%) electrophoresis and spectrophotometry (260 nm/280 nm ratio). The V3–V4 variable regions of microbial 16S rRNA genes were targeted using primer pair 338F (5′-ACTCCTACGGGAGGCAGCA-3′) and 806R (5′-GGACTACHVGGGTWTCTAAT-3′), as well as adapter sequences and barcode sequences for bacterial community analysis. PCR was performed using the following steps—initial denaturation for 5 min at 95 °C; 15 cycles of 1 min at 95 °C, annealing for 1 min at 50 °C, extension for 1 min at 72 °C; and a final extension at 72 °C for 7 min. High-throughput sequencing was conducted by Biomarker Technologies Co., Ltd. (Beijing, China), using an Illumina HiSeq 2500 (Illumina Inc., San Diego, CA, USA). After sequencing, paired-end reads were assembled with a minimum overlap of 10 bp, using fast length adjustment of short reads (FLASH; version 1.2.11). The assembled tags were compared against primers, and tags corresponding to more than six mismatches were discarded using the FASTX-Toolkit. Sequences with an average quality score greater than 20, over a 50-bp sliding window were truncated using the Trimmomatic (version 0.33). Chimeras were identified and removed using UCHIME. Subsequently, using USEARCH (version 10.0), effective sequences were clustered into operational taxonomic units (OTUs) with a similarity cutoff of 97% [16].

### 2.3. Taxonomy, Community Richness and Diversity, and Gene Function Prediction

To use most sequence data, the read counts were not rarefied to a common depth. The taxonomy of sequences was determined using the RDP classifier (version 2.2, https://sourceforge.net/projects/rdp-classifier/files/rdp-classifier/) against Silva databases (Release 128). Community richness and diversity were analyzed using alpha diversity estimators, including the abundance-based coverage estimator (ACE), Chao1, Simpson, and Shannon indexes, calculated on Mothur (version 1.30, https://www.mothur.org/) and through principal coordinates analysis (PCoA) of beta diversity using QIIME (version 1.8, https://github.com/biocore/qiime). Functions of the 16S rRNA were predicted based on the information in the Kyoto Encyclopedia of Genes and Genomes (KEGG) (http://www.genome.jp/kegg/) database. The OTU abundance table was standardized, and then the KEGG ortholog cluster information was obtained and calculated from the Greengenes ID of each out, using the PICRUSt software version 1.1.1 (http://picrust.github.io/picrust/) [17].

### 2.4. Chemical Analysis

The CODcr, 5-d biological oxygen demand (BOD5), NH_4_-N, TN, TP, suspended solids (SS), total dissolved solids (TDS), sulfide, fluoride, cyanide, zinc, iron, copper, alumina, manganese, mercury, arsenic, chromium, volatile phenol, petroleum oil, aniline, toluene, anionic surfactant, and chlorobenzene removal efficiencies of the WWTPs were analyzed. These analyses were conducted according to the corresponding standard methods [18], with assistance from the Tianjin Huanke Testing Technology Co., Ltd. (Tianjin, China), which has obtained the China Metrology Accreditation.

### 2.5. Statistical Analysis

Correlations and mean statistical differences in the experimental data were analyzed via correlation analysis and one-way analysis of variance using SPSS version 20 (IBM Corporation, Armonk, NY, USA).

## 3. Results

### 3.1. Pollutant Removal Efficiencies of the Three WWTPs

**Figure 1:**
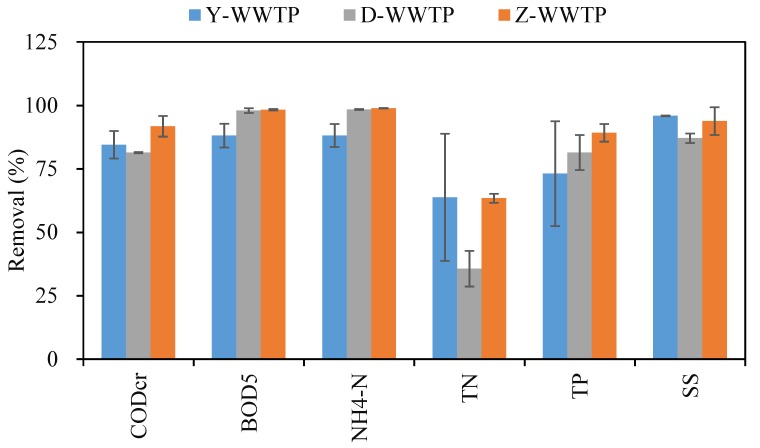
Removal efficiencies of the conventional pollutants in Y-, D-, and Z-wastewater treatment plants.

The industrial pollutants chromium, mercury, arsenic, alumina, manganese, iron, cyanide, TDS, chlorobenzene, and surfactant concentrations in the industrial influent of Y-WWTP were markedly higher than those in the municipal Z-WWTP; most of these pollutants were also significantly higher in the industrial influent of D-WWTP than the municipal influent of Z-WWTP (Table 1). The removal efficiencies for the conventional pollutants detected in the influent were lower than that of Y-WWTP at Z-WWTP (BOD_5_: *p* = 0.035; NH_4_-N: *p* = 0.023; Figure 1). Additionally, the variation of removal efficiencies of Y-WWTP was significantly larger than those of the other two WWTPs, especially for TN and TP. This indicated that the industrial influent had negative effects on the removal efficiencies of pollutants and on the treatment performance stability. If treatment by WWTPs in industrial zones was inefficient, the residual pollutants in the effluent would pose environmental risks [19].

### 3.2. Microbial Community Structure Analysis

#### 3.2.1. Microbial Community Richness and Diversity

Table 2 shows the microbial community richness and diversity indexes for all sludge samples. The Good’s estimator of coverage values for all samples were greater than 0.998, indicating that the corresponding sequence library provided complete coverage of the microbial community. Sampling location and season evidently affected microbial abundance and diversity in sludge. Sludge collected from Y-WWTP in November, termed YAW, had a significantly lower number of OTUs (*p* < 0.05) compared to those in sludge collected in November from D-WWTP (DAW) and Z-WWTP (ZAW). This indicated that industrial wastewater had considerably reduced bacterial numbers. Furthermore, YAW showed the lowest values of ACE and Chao1 indexes, which are indicators of microbial community richness. The lowest Shannon and highest Simpson indices were obtained for YAW, which are diversity indicators, suggesting that industrial wastewater had a negative impact on microbial sludge diversity. Sludge samples of WWTPs treating wastewater from industrial processes such as textile dyeing, petroleum refining, whey processing, pharmaceutical production, and pesticide production have lower microbial community richness than sludge samples of WWTPs that treat municipal wastewater [3,20,21]. The highest Shannon index was obtained for ZAW, indicating that the municipal wastewater enhanced sludge microbial diversity. Overall, the OTUs and ACE values for all sludge samples collected in September were higher than those for sludge collected in November by 8.9% and 13.5%, respectively. This suggests that bacterial community richness increases at higher temperatures (in September). Similar differences in microbial richness and diversity were observed for sludge samples from all other WWTPs. Zhang et al. (2018) also reported significantly higher microbial community richness and diversity (Shannon index) in sludge samples collected in summer than sludge collected in winter from four full-scale WWTPs [22]. Furthermore, Griffin and Wells (2017) reported that bacterial community structure in a full-scale AS process was affected by seasonal temperature fluctuations, with increased diversity at higher temperatures [23]. The abundances of several functionally important bacterial genera varied considerably with seasonal variations in temperature [24].

**Figure 2:**
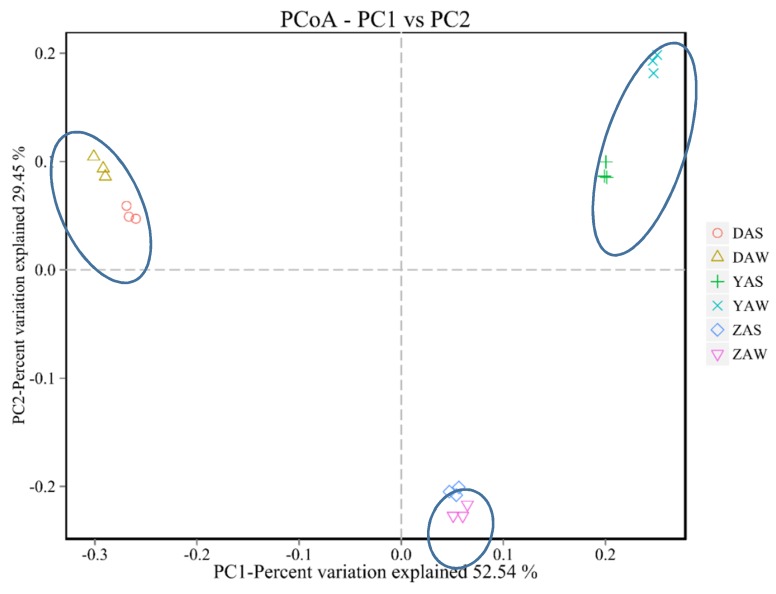
Principal coordinates analysis based on the operational taxonomic unit abundance in microbial communities of different sludge samples.

The observed differences in bacterial community structure were verified via PCoA (Figure 2). The samples, sludge collected in September from D-, Y-, and Z-WWTP (referred to as DAS, YAS, and ZAS, respectively), and in November (DAW, YAW, and ZAW), were clustered into three groups along PC1 (accounting for 52.54% of the variation). Thus, the bacterial communities of sludge from different WWTPs differed considerably. Furthermore, the microbial community structures of samples collected in November and September from the same WWTP were different, but were more similar to each other than to samples collected from other WWTPs.

#### 3.2.2. Taxonomic Classification of the Bacterial Communities

**Figure 3:**
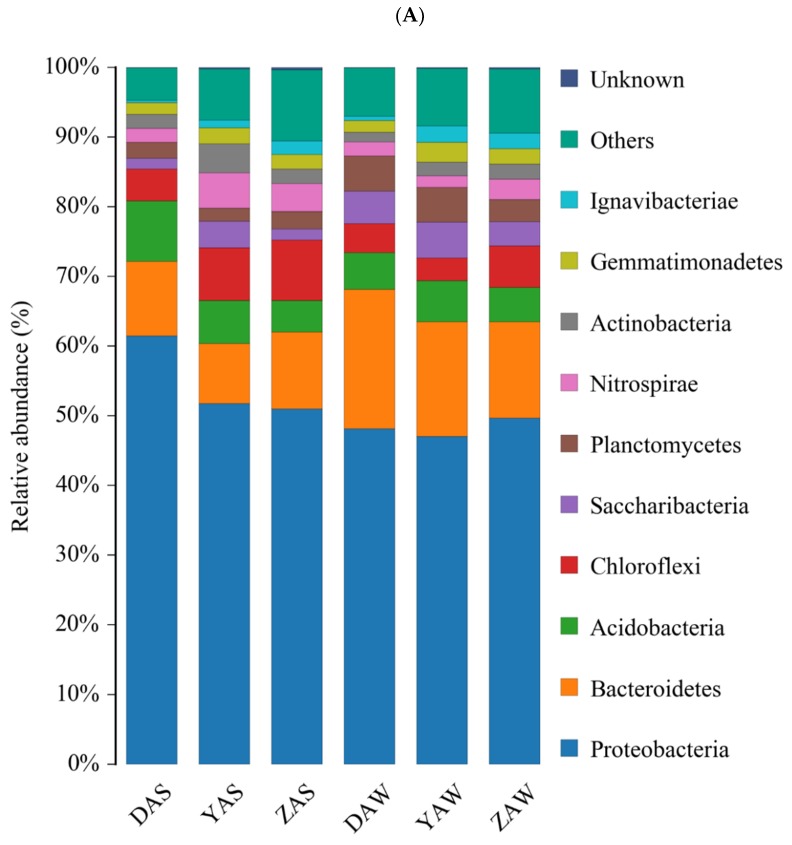

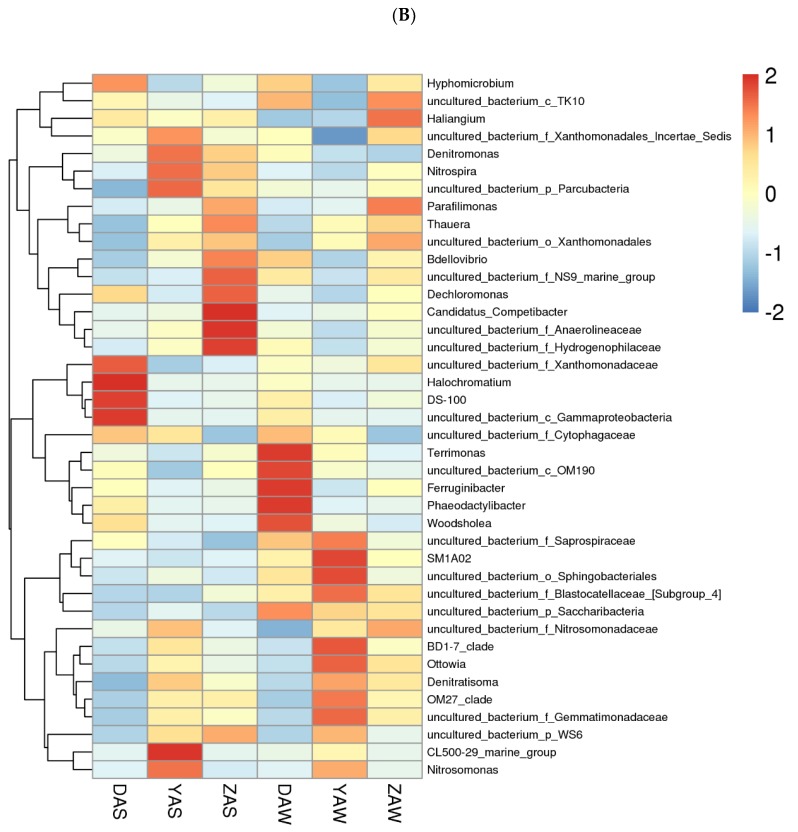
Relative abundance ((**A**) phylum level) and heat map ((**B**) genus level) of microbes in sludge samples collected from different wastewater treatment plants, at different times.

At the phylum level, 37–42 bacterial phyla were identified in the AS samples. Proteobacteria, Bacteroidetes, Acidobacteria, Chloroflexi, Saccharibacteria, Planctomycetes, and Nitrospirae were the most abundant bacteria at the phylum level in all AS samples (Figure 3A). Previous studies have shown that these bacteria play important roles in the AS processes [25,26,27]. Proteobacteria was the most dominant bacterial phylum (ranging from 47% to 52%) in the AS samples. This was consistent with a previous report that Proteobacteria was predominant in WWTPs and played a significant and broad role in organic and nutrient removal [28]. Moreover, Nitrospirae and Chloroflexi were the key bacterial phyla for nitrite oxidation and denitrification in AS [29]. The abundances of Chlamydiae, Chlorobi, Chloroflexi, Elusimicrobia, Ignavibacteriae, Latescibacteria, Parcubacteria, and Spirochaetae were the highest in ZAS (see A, Table 3). The lowest abundances (see B or C, Table 3) of Armatimonadetes, Bacteroidetes, Chlamydiae, Chlorobi, Cyanobacteria, Fibrobacteres, and Verrucomicrobia were observed for YAS, indicating an inhibition of bacterial growth by wastewater from chemical industries. Both DAS and YAS had lower Chlamydiae, Chlorobi, Chloroflexi, Elusimicrobia, Ignavibacteriae, Latescibacteria, Parcubacteria, and Spirochaetae abundances than ZAS, indicating an inhibition of these bacteria by industrial wastewater. The relative abundances of Chloroflexi and Ignavibacteriae were reported to be significantly diminished by ampicillin at 30 mg/L [25]. Among the sludge samples collected in November, the abundances of Chlorobi, Chloroflexi, Elusimicrobia, Parcubacteria, and Spirochaetae in ZAW were highest and were similar to those in ZAS. However, bacterial abundances in YAW were much lower (see C, Table 3) than those in YAS, which could be attributed to the lower shock resistance of bacteria at lower temperatures. Overall, bacterial abundance in ZAS was much higher than those in DAS or YAS.

The taxonomic classification of the microbial communities at the genus level is shown in Figure 3B. This provides more detailed information about microbial community succession. ZAS had higher abundances of *Parafilimonas*, *Thauera*, Xanthomonadales, *Dechloromonas*, *Candidatus* Competibacter, *Parafilimonas*, Anaerolineaceae, and Hydrogenophilaceae, than YAS and DAS. Furthermore, *Dechloromonas*, *Hyphomicrobium*, *Phaeodactylibacter*, *Terrimonas*, and Xanthomonadaceae abundances were the lowest in YAS, while abundances of *Denitromonas*, *Nitrosomonas*, Nitrosomonadaceae, and Saccharibacteria were the highest. ZAW had higher abundances of *Candidatus* Competibacter, *Dechloromonas*, *Haliangium*, *Nitrospira*, *Parafilimonas*, *Thauera*, Nitrosomonadaceae, Xanthomonadales, and Parcubacteria, than YAW and DAW. *Dechloromonas*, *Ferruginibacter*, *Hyphomicrobium*, *Nitrospira*, Anaerolineaceae, Hydrogenophilaceae, Xanthomonadales, and Parcubacteria were the least abundant in YAW, while *Denitratisoma*, *Nitrosomonas*, Blastocatellaceae, Saprospiraceae, and Sphingobacteriales were the most dominant. Overall, Z-WWTP had higher abundances of *Dechloromonas*, *Nitrospira*, and *Thauera*, while Y-WWTP had higher *Nitrosomonas* concentrations and lower *Nitrospira* abundances. *Dechloromonas* and *Thauera* were identified as typical denitrifying bacteria in wastewater treatment [30], while *Nitrosomonas* and *Nitrospira* were predominant ammonia oxidation bacteria and nitrite oxidation bacteria in WWTPs [31,32,33]. In an SBR process, the *Nitrospira* population decreased sharply and was ultimately wiped out in the presence of copper [34]. These dynamic changes might be attributed to the differences in wastewater type, technological process, or geographical location.

### 3.3. KEGG Analysis of Dominant Taxonomic Groups and Related Metabolic Functions

**Figure 4:**
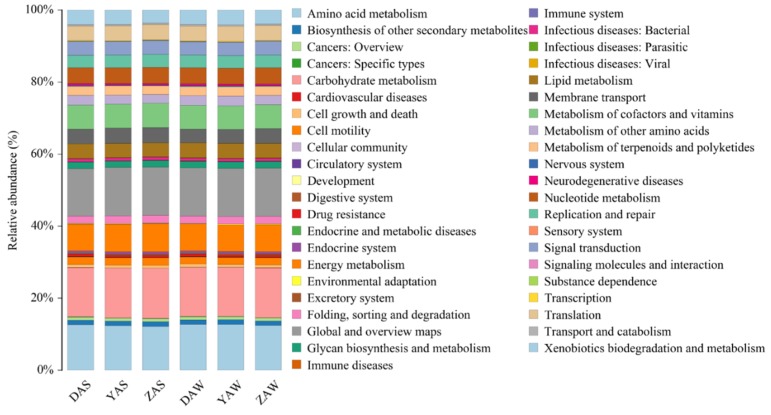
Relative abundances of genes attributable for different bacterial functions according to KEGG level 2 functional categories.

Bacterial functions predicted based on KEGG level 2 functional categories are illustrated in Figure 4. Metabolism was the dominant biochemical pathway, and the genes responsible for metabolism accounted for 74.1%–74.8% of the total, followed by genes responsible for genetic information processing (7.8%–8.0%), environmental information processing (9.7%–10.2%), human diseases (3.2%–3.4%), cellular processes (3.2%–3.4%), and organismal systems (1.3%–1.4%). The major metabolic functions were carbohydrate metabolism (13.8%), amino acid metabolism (12.5%), energy metabolism (7.5%), cofactor and vitamin metabolism (6.6%), and nucleotide metabolism (4.5%), which are all necessary metabolic activities for microbial communities [35]. Previous metagenomic studies have also reported high proportions of genes responsible for these functions in biological reactors [35,36,37,38]. Most of the metabolic functions of bacteria from different samples were highly similar (Table 4). However, xenobiotic biodegradation and metabolism, as well as amino acid metabolism, were more dominant in the sludge from industrial Y- and D-WWTP (see A, Table 4) than in municipal Z-WWTP (see B, Table 4), thereby, affecting the ability of microbes to degrade or assimilate these compounds. Samples from both November and September (Table 4) collected from D-WWTP had more genes attributable for regulating cellular processes (cell growth and death), organismal systems (environmental adaptation and nervous system), and human diseases (substance dependence and infectious diseases such as parasitic, infectious, and viral diseases, and cardiovascular disorders), which might be related to the complex compositions of the industrial influent to this WWTP. Under long-term hyper-saline stress conditions, microbial communities can develop special metabolic patterns for amino acids and membrane transporters, to maintain optimal cellular activity and removal performance [27]. Exposure to 50 mg/L of silver nanoparticles was found to significantly hinder nutrient transport and metabolism, especially amino acid transport and metabolism [39].

### 3.4. Effects of Pollutant Concentration and Operation Condition on Microbial Communities

#### 3.4.1. Pollutant Concentration

**Figure 5:**
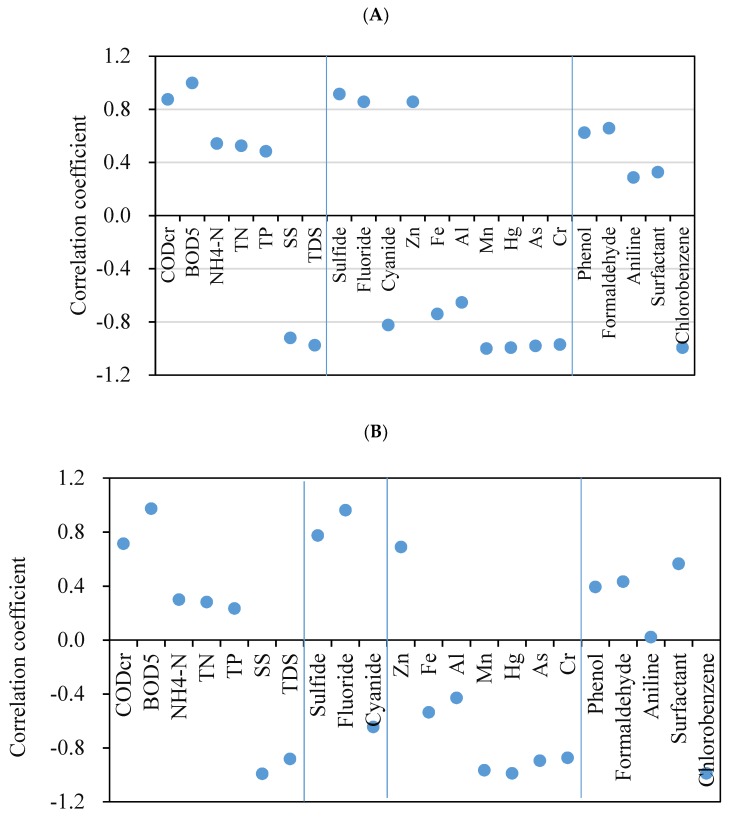

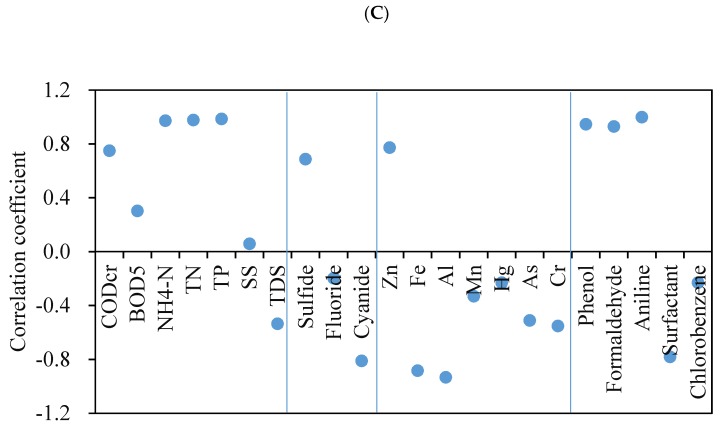
Correlation between wastewater composition and bacterial community. (**A**) Operational taxonomic unit; (**B**) ACE index; and (**C**) Shannon index.

Figure 5 shows the correlation of OTU numbers, and ACE and Shannon indices with pollutant concentration in wastewater. The OTU numbers and ACE index (Figure 5A,B), which indicate bacterial community richness, were both positively correlated with the concentration of conventional pollutants (e.g., carbon, nitrogen, and phosphorus) and negatively correlated with SS (r = 0.96) and TDS (r = 0.93). With increasing salt concentration, species richness decreased due to the selection pressure of high salt concentrations [27]. Levels of heavy metals such as mercury (r = 0.99), chromium (r = 0.92), and arsenic (r = 0.94) were negatively correlated with OTU level and ACE index, while zinc (r = 0.77) concentration showed a positive correlation with these two richness indicators. Similar phenomena have been observed in previous studies. The heavy metals mercury, arsenic, aluminum, cadmium and lead had a negative relationship with the richness of microbial communities in soil [40] and marine sediments [41]. Conversely, as an essential element for microorganism growth, several bacteria in contaminated soil were significantly positively correlated with zinc concentration [42], where low exposure to zinc ion (<5 mg/L) promoted biogas production in an anaerobic digestion process [43] and a low zinc oxide concentration enhanced the microbial richness by 21.3% in an anammox process [44]. The organic pollutants phenol (r = 0.51), formaldehyde (r = 0.55), and toluene (r = 0.39) were slightly positively correlated with bacterial species richness, whereas chlorobenzene (r = 0.99) was significantly negatively correlated. Microbial community richness and diversity decreased with continuous dosing of copper [34]. Furthermore, in this study, these pollutants had a similar effect on microbial community diversity, as indicated by the Shannon index values (Figure 5C).

#### 3.4.2. Operation Condition

Besides wastewater composition, wastewater treatment processes and operational conditions have major impacts on the microbial community richness and diversity of AS. Y-WWTP, D-WWTP, and Z-WWTP employ OD, A/O, and SBR processes, respectively, with MLSS levels of 3450–3850 mg/L, SRTs of 20–25 d, DO contents of 2.0–3.0 mg/L, and HRTs of 4.5–19.5 h. The results in this study showed significant differences in the microbial community among the three WWTPs (Figure 2 and Figure 3). It must be noted that it is difficult to qualitatively analyze the effect of operation conditions on the microbial communities in these WWTPs because they employ different systems. Previous studies have shown that the MLSS concentration is correlated with the abundances of certain microorganisms [45]. Furthermore, a high HRT favored growth of certain filamentous bacteria in membrane tank reactors, thereby, changing the bacterial community [46]. By contrast, a shorter HRT supported fewer microbial species, which in turn resulted in the utilization of fewer carbon sources [47]. However, no significant correlation was noted between bacteria genera number and DO content during municipal wastewater treatment [48]. Finally, temperature had significant effects on nitrification performance in a denitrification biofilter through the change of ammonia-oxidizing bacteria [49]. The microbial communities and treatment performances of centralized WWTPs in industrial zones were negatively affected by the industry-related pollutant loading. Therefore, it is important to enhance the shock resilience to obtain an optimal microbial community structure for high efficiency of wastewater treatment, by selecting anti-shock loading treatment processes; increasing the returning sludge; and optimizing the extended aeration and chemical addition in the centralized WWTPs.

## 4. Conclusions

We investigated microbial community structure, diversity, and functions in industrial and municipal WWTPs. Sludge from Y-WWTP (processing chemical industrial wastewater) had significantly lower OTU levels and ACE, Chao1, and Shannon indices (*p* < 0.05) than sludge from D-WWTP (processing comprehensive industrial wastewater) and municipal Z-WWTP, indicating that chemical industrial wastewater significantly inhibited bacterial richness and diversity. Furthermore, bacterial richness was positively correlated with conventional pollutants (e.g., carbon, nitrogen, and phosphorus), but negatively correlated with TDS. Influent composition affected the abundances of nitrifying and denitrifying microbes (e.g., *Dechloromonas*, *Nitrospira*, *Nitrosomonas*, and *Thauera*) and xenobiotic degradation by microbes. These results provide support for the control of wastewater effluent from upstream enterprises, and the strengthening of treatment performance of downstream centralized WWTPs in industrial zones.

## Figures and Tables

**Table 1:** Composition and concentration of raw sewage in the tested wastewater treatment plant (WWTPs).

Pollutants	D-WWTP	Y-WWTP	Z-WWTP
CODcr (mg/L)	185.17	221.00	234.00
BOD5 (mg/L)	47.87	49.64	41.80
NH_4_-N (mg/L)	13.87	26.28	36.60
TN (mg/L)	19.20	39.81	45.60
TP (mg/L)	2.40	3.95	4.36
Suspended solids (mg/L)	45.67	88.17	63.00
Total dissolved solids (mg/L)	2578.33	3748.33	770.00
Sulfide (mg/L)	3.322	0.922	8.860
Fluoride (mg/L)	1.170	0.882	1.080
Cyanide (μg/L)	4.3	61.7	4.0
Zn (mg/L)	0.192	0.126	0.428
Fe (mg/L)	3.128	3.329	0.622
Al (mg/L)	1.413	1.519	0.172
Mn (mg/L)	0.293	0.323	0.180
Hg (μg/L)	0.038	0.248	0.040
As (μg/L)	0.600	0.783	0.500
Cr (mg/L)	0.030	0.060	0.004
Phenol (mg/L)	0.014	0.015	0.028
Formaldehyde (mg/L)	0.057	0.050	0.140
Aniline (mg/L)	0.082	0.062	0.220
Surfactant (mg/L)	0.834	0.473	0.170
Chlorobenzene (μg/L)	1.000	159.867	1.000

**Table 2:** Microbial community richness and diversity indices for sludge samples collected from different wastewater treatment plants.

Sampling Date	Sludge Sampling Site	OTU	ACE	Chao1	Shannon	Simpson
	D-WWTP	873 ^a^	956 ^a^	985 ^a^	5.694 ^b^	0.007 ^c^
November.	Y-WWTP	759 ^b^	864 ^b^	871 ^b^	5.344 ^c^	0.010 ^a^
	Z-WWTP	888 ^a^	945 ^a^	957 ^a^	5.763 ^a^	0.008 ^b^
	D-WWTP	962 ^a^	1010 ^a^	1020 ^a^	5.271 ^c^	0.024 ^a^
September.	Y-WWTP	921 ^b^	989 ^a^	1022 ^a^	5.372 ^b^	0.011 ^b^
	Z-WWTP	968 ^a^	1007 ^a^	1017 ^a^	5.612 ^a^	0.010 ^b^

^a^, ^b^, and ^c^: multiple mean comparisons using alphabetic labels, where a > b > c. Different letters indicate significant differences at 0.05.

**Table 3:** Multiple mean comparison of bacterial abundance at the phylum level (significance level of 0.05).

Bacteria	Sludge Sample (Summer)			Sludge Sample (Winter)		
	DAS	YAS	ZAS	DAW	YAW	ZAW
Acidobacteria	A	B	C	AB	A	B
Actinobacteria	B	A	B	B	A	A
Aminicenantes	B	A	B	A	A	A
Armatimonadetes	A	C	B	A	B	B
BRC1	B	A	A	B	A	B
Bacteroidetes	A	B	A	A	B	C
Chlamydiae	B	C	A	A	C	B
Chlorobi	B	C	A	B	C	A
Chloroflexi	C	B	A	B	C	A
Cyanobacteria	A	C	B	A	C	B
Deferribacteres	A	B	B	A	A	A
Elusimicrobia	B	B	A	B	B	A
Fibrobacteres	A	B	A	A	C	B
Firmicutes	C	A	B	B	B	A
Fusobacteria	A	A	A	A	A	A
Gemmatimonadetes	B	A	A	C	A	B
Gracilibacteria	A	A	B	A	C	B
Hydrogenedentes	A	B	B	A	B	B
Ignavibacteriae	C	B	A	B	A	A
Latescibacteria	B	B	A	A	A	B
Microgenomates	A	B	B	A	B	B
Nitrospirae	C	A	B	B	C	A
Parcubacteria	C	B	A	B	C	A
Peregrinibacteria	A	A	A	B	A	B
Planctomycetes	AB	B	A	A	A	B
Proteobacteria	A	B	C	B	C	A
RBG-1[Zixibacteria]	B	B	A	B	B	A
SR1[Absconditabacteria]	B	B	A	A	A	A
Saccharibacteria	B	A	B	B	A	C
Spirochaetae	B	B	A	B	C	A
Synergistetes	A	A	A	A	A	A
TM6[Dependentiae]	A	C	B	A	AB	B
Verrucomicrobia	A	B	A	A	A	A

A, B, and C—multiple mean comparisons using alphabetical labels, where A > B > C. Different letters indicate significant differences at 0.05.

**Table 4:** Multiple mean comparison of gene abundance according to bacterial functions in different sludge samples based on KEGG level 2 categories.

Class 1	Class 2	DAS	YAS	ZAS	DAW	YAW	ZAW
Metabolism	Carbohydrate metabolism	A	A	A	A	A	A
Metabolism	Lipid metabolism	A	A	A	A	A	A
Metabolism	Metabolism of cofactors and vitamins	A	A	A	A	A	A
Metabolism	Energy metabolism	A	A	A	A	A	A
Metabolism	Nucleotide metabolism	A	A	A	A	A	A
Metabolism	Biosynthesis of other secondary metabolites	A	A	A	A	A	A
Metabolism	Metabolism of terpenoids and polyketides	A	A	A	A	A	A
Metabolism	Glycan biosynthesis and metabolism	AB	B	A	A	A	A
Metabolism	Global and overview maps	A	A	A	A	A	A
Metabolism	Amino acid metabolism	A	A	A	A	AB	B
Metabolism	Xenobiotics biodegradation and metabolism	A	A	B	A	A	B
Metabolism	Metabolism of other amino acids	A	AB	B	A	AB	B
Environmental Information Processing	Membrane transport	A	A	A	A	A	A
Environmental Information Processing	Signal transduction	A	A	A	A	A	A
Cellular Processes	Cell motility	A	A	A	A	A	A
Cellular Processes	Transport and catabolism	A	A	A	A	A	A
Cellular Processes	Cell growth and death	A	B	B	A	A	B
Cellular Processes	Cellular community	A	B	C	AB	B	A
Genetic Information Processing	Folding, sorting and degradation	B	AB	A	A	A	A
Genetic Information Processing	Transcription	A	A	A	A	A	A
Genetic Information Processing	Translation	A	A	A	A	A	A
Genetic Information Processing	Replication and repair	A	A	A	A	A	A
Organismal Systems	Endocrine system	A	A	A	AB	A	B
Organismal Systems	Circulatory system	A	A	A	A	A	A
Organismal Systems	Immune system	B	B	A	A	A	A
Organismal Systems	Environmental adaptation	A	B	B	A	AB	B
Organismal Systems	Nervous system	A	B	B	A	A	B
Organismal Systems	Sensory system	A	B	B	A	B	A
Organismal Systems	Excretory system	B	B	A	AB	A	B
Organismal Systems	Digestive system	A	B	AB	A	A	B
Human Diseases	Drug resistance	A	A	A	A	AB	B
Human Diseases	Endocrine and metabolic diseases	A	A	A	A	A	A
Human Diseases	Cancers: Overview	A	A	A	A	A	A
Human Diseases	Infectious diseases: Bacterial	A	A	A	A	A	A
Human Diseases	Neurodegenerative diseases	A	B	B	A	AB	B
Human Diseases	Substance dependence	A	B	B	A	B	C
Human Diseases	Infectious diseases: Parasitic	A	C	B	A	B	B
Human Diseases	Infectious diseases: Viral	A	B	B	A	B	B
Human Diseases	Cancers: Specific types	A	B	B	A	A	A
Human Diseases	Immune diseases	A	B	B	A	A	B
Human Diseases	Cardiovascular diseases	A	B	C	A	B	B

A, B, and C—multiple mean comparisons using alphabetical labels, where A > B > C. Different letters indicate significant differences at 0.05.
